# Editorial: Focus on *Popillia japonica*: New research for IPM of the Japanese beetle

**DOI:** 10.3389/finsc.2024.1532825

**Published:** 2025-01-24

**Authors:** Giselher Grabenweger, Giulia Torrini, William D. Hutchison

**Affiliations:** ^1^ Extension Arable Crops, Department of Plants and Plant Products, Agroscope, Zurich, Switzerland; ^2^ CREA - Research Center for Plant Protection and Certification, Florence, Italy; ^3^ Department of Entomology, University of Minnesota, St. Paul, MN, United States

**Keywords:** *Popillia japonica*, Japanese beetle, invasive pest, management, editorial

The arrival of the Japanese Beetle, *Popillia japonica* Newman, in continental Europe (1) has sparked new interest in this well-known invasive species, and initiated international collaboration for its control, not only within Europe but also across the Atlantic. A session dedicated to this invasive pest at the International Congress of Entomology in Helsinki in 2022 enabled exchange between leading *Popillia* experts from the US and Canada and scientists involved in the containment of the Japanese Beetle in Europe. The most important work presented during this session is compiled in the presented e-book, along with other research updates regarding the biology, ecology and management of *P. japonica*, which was not included in previous reviews about this pest (2–4).

In this Research Topic, entitled “*Focus on Popillia japonica*,” two articles deal with more robust estimates of *P. japonica* damage to important crops like wine grapes. Ebbenga et al. estimates the impact of *P. japonica* infestation on yield and grape quality. Although it is long known that grapes (cultivated and wild) is one of the beetle’s preferred host plants, this paper is the first to assess the damage and impact on several berry/juice quality parameters. Straubinger et al. carried out a survey among Italian wine growers and attempted to put a price tag on the *P. japonica* invasion into the Piedmont wine growing region. Their findings show that increasing labor costs as a consequence of the invasion are responsible for about two thirds of the farmers’ loss, while yield loss and costs of the plant protection treatments themselves are less important.

Investigations on the spatial distribution of the pest in certain crops as well on a wider scale are the topic of another two contributions. Henden and Guédot investigated how geographic, climatic, and landscape factors influence the spatial distribution of *P. japonica* abundance. They found that the abundance of Japanese beetles was higher in vineyards with pastures in the surrounding landscape, with higher temperatures, and located further east in the area of Southern Wisconsin. High leaf damage occurred at similar sites, but only when pesticide use was low. A tool that comes in handy in IPM against *P. japonica* was developed by (Toninato et al.). With their sequential sampling plan, farmers can estimate a *P. japonica* population density in raspberry fields, and consequently get a robust basis for a control decision, within an inspection time of about 11minutes per site only. They also could show that spatial patterns of the Japanese beetle abundance are not influenced by the use of insecticides, at least in cases where aggregation behavior may be triggered by stronger factors like host plant preference.

Alternatives to time-consuming monitoring of *P. japonica* with lure traps are presented in the following articles. Ribeiro et al. tested the feasibility of remote sensing for the detection of *P. japonica* in soybean. They found evidence that injury on leaves caused by *P. japonica* tends to reduce soybean canopy reflectance at wavelengths of 700-1000 nm. Such technology may come in handy for pest detection on large fields. Another paper by Ebbenga et al. describes a degree-day model to forecast flight periods of adult Japanese beetles. They were able to develop a straight forward model by summarizing degree-days starting from January 1^st^ and setting lower and upper thresholds of 15 and 21.7°C, respectively. Upon reaching 257 and 345 degree-days, respectively, 10% and 50% of *P. japonica* adult emergence is forecasted to be underway.

Two contributions focus on the current invasion of *P. japonica* in continental Europe. Gotta et al. describe the many control attempts undertaken by Italian authorities and producers since the first detection of the pest in northern Italy in 2014. They review the strengths and weaknesses of chemical, physical, and biological control measures deployed for containment of *P. japonica* in the infested zones of the Lombardy and the Piedmont region. Poggi et al. performed a pest risk analysis for the pest-free (to date) region of Metropolitan France, which is quite close to infested regions geographically and also well-connected with infested zones by major routes of transport of humans and goods. They recommend early detection and early-stage eradication measures against *P. japonica* in outbreak zones as the most important measure to control the risk of pest invasion.

The development of environmentally friendly control measures against the invasive pest is the main goal of the last two contributions to this e-book. Graf et al. present experiments for biological control of *P. japonica* with entomopathogenic fungi (EPF). Their study gives evidence that Japanese beetle larvae are resistant to EPF infection, while adults are very susceptible. Consequently, more resources should be invested into the control of adult *P. japonica* with EPF. Carroll et al. tested gene silencing by feeding *P. japonica* with double-stranded RNA as a novel control approach. They show that fast degradation of dsRNA in the insect’s gut may be avoided by micro-encapsulation, which increases gene knock-down and, consequently, efficacy of the control approach against the invasive pest.

As you read this summary, *P. japonica* continues to expand its range, slowly but steadily, in the US and Canada as well as in Italy and Switzerland, bringing new challenges to producers as well as plant health specialists on both continents. It is clear that single control measures will never stop this invasion, and that elaborate IPM strategies are necessary for successful *P. japonica* containment. We are hopeful that the contributions compiled in this e-book will contribute to the development of a sustainable response to the ongoing invasion.
